# Evaluating the comorbidities of age and cigarette smoking on stroke outcomes in the context of anti-complement mitigation strategies

**DOI:** 10.3389/fimmu.2023.1161051

**Published:** 2023-05-08

**Authors:** Christine Couch, Ali M. Alawieh, Amer Toutonji, Carl Atkinson, Stephen Tomlinson

**Affiliations:** ^1^ Department of Microbiology and Immunology, Medical University of South Carolina, Charleston, SC, United States; ^2^ Department of Neurosurgery, Emory University School of Medicine, Atlanta, GA, United States; ^3^ Division of Pulmonary, Critical Care and Sleep Medicine, University of Florida, Gainesville, FL, United States; ^4^ Ralph H. Johnson Veteran's Affairs (VA) Medical Center, Charleston, SC, United States

**Keywords:** complement, stroke, cigarette smoke, age, comorbidity

## Abstract

Multiple neuroprotective agents have shown beneficial effects in rodent models of stroke, but they have failed to translate in the clinic. In this perspective, we consider that a likely explanation for this failure, at least in part, is that there has been inadequate assessment of functional outcomes in preclinical stroke models, as well the use of young healthy animals that are not representative of clinical cohorts. Although the impact of older age and cigarette smoking comorbidities on stroke outcomes is well documented clinically, the impact of these (and other) stroke comorbidities on the neuroinflammatory response after stroke, as well as the response to neuroprotective agents, remains largely unexplored. We have shown that a complement inhibitor (B4Crry), that targets specifically to the ischemic penumbra and inhibits complement activation, reduces neuroinflammation and improves outcomes following murine ischemic stroke. For this perspective, we discuss the impact of age and smoking comorbidities on outcomes after stroke, and we experimentally assess whether increased complement activation contributes to worsened acute outcomes with these comorbidities. We found that the pro-inflammatory effects of aging and smoking contribute to worse stroke outcomes, and these effects are mitigated by complement inhibition.

## Introduction

Acute ischemic stroke occurs secondary to thrombosis or embolization within the cerebral vasculature, which leads to an infarct within the brain and clinical deficits. The standard of care for stroke therapy is rapid recanalization of the target vessel, either pharmacologically or endovascularly. Over the past decade, outcomes following acute stroke have improved significantly due to both stroke prevention efforts and the introduction of endovascular thrombectomy as the routine standard of care. Nevertheless, stroke remains a major cause of disability and mortality in the United States and Worldwide. The current standard of care for stroke patients remains rapid reperfusion using thrombolysis and/or thrombectomy for eligible patients that present within about 24 hours of onset (1–3). However, despite a successful recanalization rate of over 85%, the rate of functional independence at 90 days remained at less than 50% in successfully recanalized patients (3, 4). Reasons for this mismatch between recanalization and recovery are multiple and include the rapid progression of infarct secondary to inflammation, microthrombosis in the microvasculature, hemorrhagic complications, and limited rehabilitation support. Several clinical studies have focused on identifying a subset of stroke patients that are termed “fast progressors”, that is patients whose infarct progresses rapidly despite recanalization and who tend to have worse functional outcomes. Poor cerebrovascular reserve and collateral circulation are considered major culprits within this patient group, which is attributed to advanced age and other comorbidities, as well as an enhanced local neuroinflammatory response. Data from thrombectomy trials lend strong support to the concept that neuroprotective adjuvant therapies will leverage the benefit of reperfusion and limit the progression of cerebral tissue loss after stroke. However, there are currently no neuroprotective agents approved for ischemic stroke, with multiple agents having failed in clinical trials. This failure of neuroprotective agents is multifactorial, but one perspective is that this is due in large part to the poor design of preclinical studies that often lacked consideration of long-term outcomes, cognitive recovery, rehabilitative interventions, and relevant to this perspective article, stroke comorbidities (5, 6). The translational importance of incorporating stroke comorbidities in evaluating preclinical efficacy of neuroprotective and anti-inflammatory therapies for stroke is being increasingly recognized. Within this context, previous studies from our lab and others have studied the role of the complement system in initiating and propagating a neuroinflammatory response after stroke (4, 7–10),, and inhibition of complement has been shown to provide long-lasting neuroprotection in murine stroke models. However, these previous investigations were almost exclusively performed using healthy young adult mice. Complement inhibitors are recognized as potential therapeutic agents for treating stroke, and here we provide an assessment on the effects of two major stroke comorbidities, namely advanced age and cigarette smoking (CS), within the framework of complement-dependent neuroinflammation and recovery.

## Impact of cigarette smoking and aging in ischemic stroke

Comorbidities in patients present as a cluster of risk factors that increase stroke incidence. What is much less appreciated is that comorbidities also alter stroke pathophysiology, lesion development and recovery in profound ways. With the use of genetic animal models and/or pharmacological interventions, it is possible to capture certain features of comorbidities. Comprehensive reviews on animal models with comorbidities are available elsewhere (11, 12).

Cigarette smoke is a patient modifiable risk factor for ischemic stroke, and is correlated with an increased risk of mortality, more severe disability, longer hospital stays and worse overall functional recovery (13, 14). Cigarette smoking nearly doubles the risk for stroke, with a dose response relationship between pack-years and stroke risk (15, 16). Age is also recognized as a significant predictor of stroke outcome, affecting speed and extent of recovery, mortality, and response to thrombolytic therapy (17). However, the mechanisms underlying how either comorbidity contributes to worse outcomes are not well understood, and there are very few reports on neuroprotective therapies in the context of these comorbidities, despite continuous recommendations from the STAIR committee and funding bodies. There has only been a single report investigating the effects of cigarette smoke on in the brain, in which it was shown that cigarette smoke exposure induced activation of inflammatory cascades and increased oxidative stress (18). In other (non-stroke) models, our lab and others have shown that cigarette smoke is associated with altered systemic inflammatory profiles, including complement activation (19–22). Smoking also contributes to decreased vessel wall integrity (19) and may be associated with increased risk of hemorrhagic transformation or intracranial hemorrhage after thrombolytic therapy. There are also only very few reports on the consequences of aging on post stroke neuroinflammation, even though with increasing life expectancy, aging has become a principal risk factor for stroke. It is known that in the absence of ischemic pathology, the aging brain shows a gradual increase in inflammatory signaling (22) and an increase in reactive oxygen species both basally and in response to injury (23, 24). In the normal ageing brain, there is also increased expression of innate immune molecules, including complement proteins (25). However, as with CS exposure, the role of aging in the context of neuroinflammation and neuroprotection after stroke remains poorly investigated.

## Age and CS-exposure lead to worse acute outcomes after murine stroke, an effect that is reversed with complement inhibition

To investigate how age and CS affect a complement-dependent neuroinflammatory response and behavioral outcomes after stroke, we utilized a murine model of 60 minute transient middle cerebral artery occlusion (MCAO) and the site-targeted complement inhibitor, B4Crry, as previously described (9). The B4Crry inhibitor targets specifically to the ischemic and perilesional region of the post-ischemic brain after MCAO and locally inhibits all complement pathways at the C3 activation step and blocks the generation of both C3 opsonins and C3a (9). At the dose used in the studies reported here, B4Crry has no effect on systemic (blood) complement activity (9).

In an initial study, we exposed mice to 6 months of CS (see supplement) starting at 6-8 weeks of age. Following CS exposure, mice were randomized into vehicle (PBS) or B4Crry treatment groups, and treatment administered 2 hours post MCAO (1 h post-reperfusion). No animals in the CS exposed PBS treated group survived beyond 24 h post-MCAO, whereas 40% of B4Crry treated mice survived to 6 days post-MCAO (n = 10). For this reason, we switched to a 4 month CS exposure paradigm which resulted in improved 24 hour mortality rates (see below).

**Figure 1:**
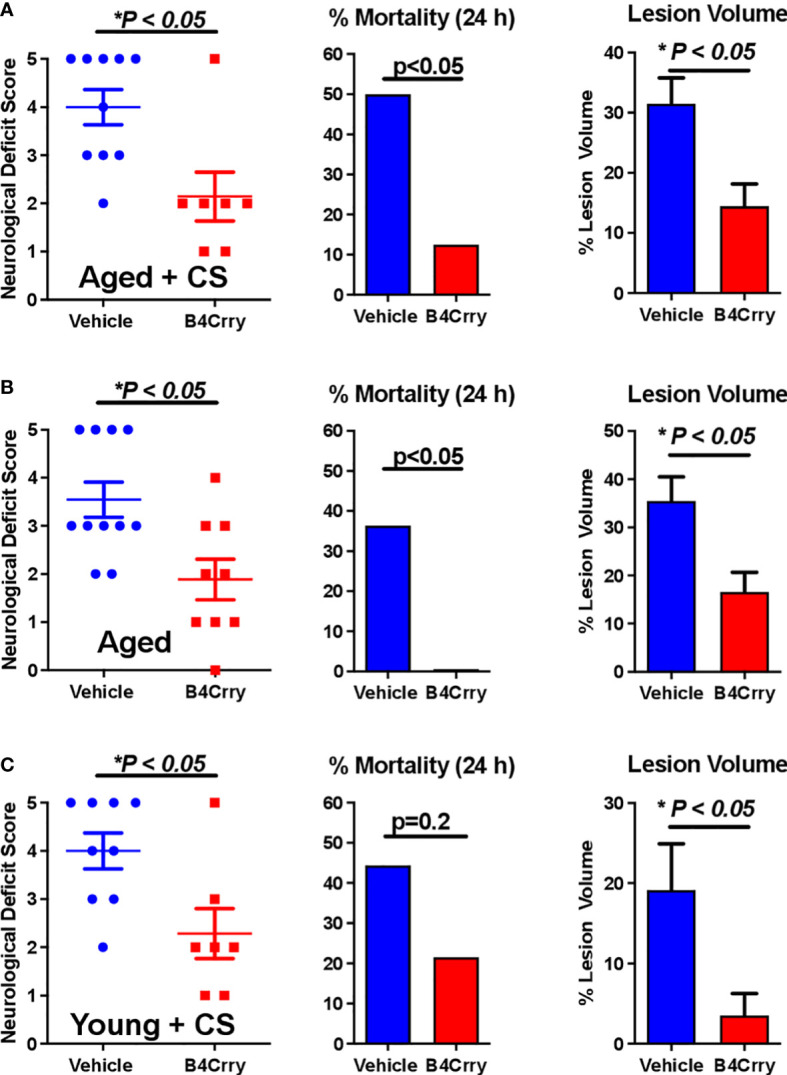
Effect of B4-Crry treatment on neurological deficit, mortality and lesion volume 24 hours after MCAO in aged and CS exposed mice. **(A)** Aged + CS exposed mice. **(B)** Aged non-CS exposed mice. **(C)** Young + CS exposed mice. Neurological deficit scores, Mann Whitney test. Mortality, Chi squared test. Lesion Volume, T-test. For all groups, n=7-11. Error bars = SEM.* indicate P value less than 5.

We assessed the impact of age and CS-exposure on acute outcomes following transient MCAO in terms of neurological deficit, mortality and infarct volume. As above, mice were randomized into either vehicle or B4Crry treatment groups. B4Crry or vehicle was administered 2 hours post MCAO to aged + CS-exposed mice, young + CS-exposed or aged room air mice. As noted above, CS exposure was for 4 months. Our previously published data using young adult mice and the same MCAO model showed a mortality rate of less than 10% at 24 hour post-MCAO (9). Here we show that aged + CS exposed mice had a mortality rate of 50%, and aged mice a mortality rate of 35% at 24 hours after stroke (**Figures 1A, B**). Thus, the impact of CS on mortality in aged mice was higher than that of aging alone. Complement inhibition with B4Crry resulted in a significant reduction in mortality rates at 24 hours in both aged and aged + CS-exposed mice. B4Crry treatment also resulted in a significant reduction in neurological deficit scores and in lesion volume in both groups compared to vehicle (**Figures 1A, B**). There was no significant difference in 24 hour survival between vehicle and B4Crry treated mice that were exposed to CS. However, B4Crry treatment did significantly improve neurological deficit scores and reduced lesion volume (**Figure 1C**). To determine any effect of aging on outcomes of CS exposed mice, aged + CS (**Figure 1A**) vs. young + CS (**Figure 1C**) outcomes were compared. There was no difference in neurological deficit score or mortality (p>0.05), although the younger animals had a significantly reduced lesion volume (p<0.05). There was also significantly reduced mortality and smaller lesion volumes in young compared to aged B4Crry treated animals (p<0.05). When the effect size of B4Crry on neurological outcome was computed, the protective effect of B4Crry was highest in aged + CS-exposed mice (Cohen’s d index 1.50), followed by CS-exposed young mice (Cohen’s d index 1.36), followed by aged no CS mice (Cohen’s d index 1.21). These findings indicate that the effects of age and CS exposure on neurological outcome after MCAO is at least in part mediated by complement.

## Complement inhibition reduces the extent of dendritic loss and microglial activation in aged mice and aged+cigarette exposed mice

**Figure 2:**
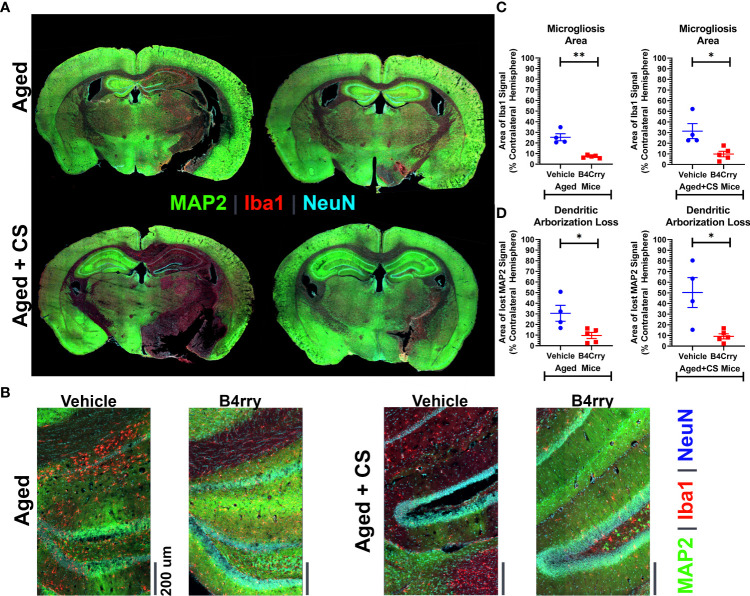
Extent of Microgliosis and loss of dendritic arborization at 24 hours after MCAO in the perilesional area of B4-Crry and vehicle treated CS exposed and non-CS exposed aged mice. **(A, B)** Immunofluorescence staining for MAP2 (dendritic marker, green), microglia (Iba1, red), and neurons (NeuN, cyan) demonstrating wider extent of microgliosis and dendritic loss in vehicle treated compared to B4Crry treated mice. Representative images. **(C)** Quantification of images in **(B)** showing percentage area of microgliosis in the ipsilateral hemisphere. Student’s T-test. *p<0.05. **p<0.01. Mean +/- SEM. **(D)** Quantification of images in **(B)** showing percentage area with loss of MAP2 signal indicating loss of dendritic arborization in the ipsilateral hemisphere in aged mice (left) and aged + CS exposed mice (right), comparing vehicle to B4Crry treatment. Student’s t-test. *p<0.05. Mean +/- SEM.

To assess how CS-exposure affects a complement mediated post-stroke neurodegenerative neuroinflammatory response in aged mice, the extent of dendritic loss and microgliosis 24 hours after MCAO in the context of B4Crry treatment was assessed. This was achieved by high resolution immunoflurescence imaging of MAP2 (dendritic marker), microglia/macrophages (Iba1) and neurons (NeuN) (**Figures 2A, B**). Unbiased stereology was used for quantification (**Figures 2C, D**). Compared to vehicle treated controls, B4Crry treatment significantly reduced microgliosis and preserved dendritic signal in the ipsilateral hemisphere in both aged and aged-CS exposed mice. There was no significant difference in the extent of microgliosis or dendritic loss between aged and aged + CS exposed mice.

## Discussion and conclusions

The failure of neuroprotective agents in stroke clinical trials is multifactorial, but one contributing factor is thought to be a lack of accounting of stroke comorbidities when evaluating drugs in preclinical studies. A neuroinflammatory response that occurs after stroke is considered to be a major contributor to secondary injury after stroke, and we investigated whether the complement system, a central component of a neuroinflammatory response after stroke, is involved in the negative effects of stroke comorbidities on stroke outcomes. Specifically, we addressed age, a non-modifiable stroke risk factor, and CS exposure, a modifiable risk factor. Both cigarette smoking and aging are factors known to affect mortality and disability after stroke (13, 14, 26). However, the mechanisms underlying the effects of age and CS exposure on stroke outcomes are poorly understood, with limited reports on neuroprotective therapies in the context of either cigarette smoking and aging, despite continuous recommendations from the Stroke Treatment Academic Industry Roundtable (STAIR) committee. We investigated acute inflammatory profiles and outcomes in aged mice, and CS exposed aged and young mice in the context of complement inhibition.

Consistent with prior reports describing a more systemic proinflammatory phenotype associated with CS (19, 21, 27), we demonstrate that CS is associated with increased local complement activation after stroke. In the aging brain, increased local complement activity is documented in the presence or absence of an ischemic or traumatic event (22–25, 28, 29). Our previous studies using murine models of ischemic stroke have demonstrated that local complement activation in the post-ischemic brain plays an important role in rapid neurological cell loss and worsening outcomes (4, 9, 10, 30, 31). We have shown that MCAO promotes neuronal stress in perilesional areas and that stressed, but still viable neurons, display danger associated molecular patterns (DAMPs) that activate complement leading to aberrant neuronal uptake by microglia (9). Here we show that after MCAO, the stroke comorbidities of age and CS exposure exacerbate both infarct growth and a neuroinflammatory response. Local inhibition of complement with B4Crry interrupted this response and reversed the effects of age and CS exposure on acute progression of a neurodegenerative inflammatory response.

The findings presented support the hypothesis that the impact of stroke comorbidities on worsening stroke outcomes, and specifically the comorbidities of age and CS exposure, is due at least in part to a complement-mediated neuroinflammatory response. Today, patients who present with a high burden of infarcted brain as measured by perfusion imaging are not eligible for endovascular intervention due to risk of hemorrhage and worsening edema (32–35). In this context, a complement inhibitor has the potential for delaying the progression of infarct in patients en route to a comprehensive stroke center for endovascular surgery, as well as for minimizing post-interventional cerebral edema and hemorrhage risk. Both effects would likely increase the subset of stroke patients eligible for intervention and lead to improved functional outcomes. A key message from this work is the implication that complement-mediated neuroinflammation is a major contributor to exacerbation of cerebral injury after stroke, and that this complement-mediated effect is more prominent in subjects with stroke comorbidities. Using pharmacological interventions that can limit the progression of infarct and temporarily preserve the penumbra remains an unmet clinical need, and one that complement inhibition shows potential for fulfilling.

In summary, this perspective supports the narrative that the inclusion of comorbidities in experimental stroke models is important to more accurately represent the stroke patient population, and which is necessary to create neuroprotective therapeutics with a higher level of success in human clinical trials. In this report, we show that complement inhibition is neuroprotective in a model that includes the comorbidities of age and CS exposure, suggesting a improved likelihood for successful translation to a heterogeneous human stroke population.

## Data availability statement

The original contributions presented in the study are included in the article/**Supplementary Material**. Further inquiries can be directed to the corresponding authors.

## Ethics statement

All animal studies were approved by the Institutional Animal Care and Use Committee (IACUC) at the Medical University of South Carolina.

## Author contributions

AA, CA and ST conceived and planned the reported studies. CC, AA, AT performed the experiments. All authors contributed to the article and approved the submitted version. CC, AA and ST formulated the perspective. ST acquired funding for the study.
