# Supplementary material for: Evaluating the comorbidities of age and cigarette smoking on stroke outcomes in the context of anti-complement mitigation strategies
Source: Front Immunol. 2023 May 8;14:1161051. doi: 10.3389/fimmu.2023.1161051 (PMC10200924; doi:10.3389/fimmu.2023.1161051)
Supplement: Supplementary file 1 [file DataSheet_1.pdf]

SUPPLEMENTAL MATERIAL

Materials and Methods

Study Design

For all studies, before the initial acclimation on behavior tasks, male mice were randomly assigned to treatment groups using a random number generator. Lab personnel involved in surgeries, testing, and scoring were blinded to group allocations for the duration of the study. Animals were excluded if mortality occurred during surgery or before administration of treatment, as well as if there was less than an 80% reduction in ipsilateral cerebral blood flow compared with presurgical baseline (see below). Both young adult and aged mice were sacrificed 24 hours after transient MCAO, at which time brain infarct volume and immunofluorescence (IF) parameters were measured. Neurological deficit scoring was measured at 24 hours after MCAO, just prior to sacrifice. End points were determined by time after reperfusion and were 24 hours, or for humane reasons as defined by institutional guidelines. Well defined humane and experimental endpoints were developed through a collaboration with our veterinarian and a scoring matrix was used to determine when pre-emptive euthanasia was necessary.

Recombinant Proteins

The recombinant complement inhibitor B4-Crry used in this study was constructed, expressed, purified, and subjected to quality control as described previously (10), (36).

The MCAO model and Treatment Paradigm

Male adult C57BL/6J mice were obtained from The Jackson Laboratory and allowed 1 week of acclimation before use. Adult animals were 6 months old and aged animals were 16 months old at the time of stroke surgeries. Anesthesia and transient MCAO was performed as described previously (10). Cerebral blood flow was assessed at baseline (just before MCAO procedure) and during MCA occlusion, as well as blood pressure and heart rate monitored. Laser Doppler flow monitoring (moorVMS-LDF1 device; Moor Instruments) was used to assess uniform induction of ischemia across animals. Animals with less than 80% reduction in ipsilateral cerebral blood flow compared with presurgical baseline were excluded from study. After surgery and during recovery from anesthesia, temperature was maintained at 37°C and animals were housed in a temperature and humidity-controlled chamber until recovery from anesthesia, and then returned to regular housing. B4Crry (16mg/kg) or vehicle (PBS) was administered intravenously via tail vein injection at 2 hours post-MCAO. We have previously performed dose-response experiments with B4Crry and have shown optimal benefit plateaus between 8-16 mg/ml and is an effective dose in both adult and aged male mice (9). All animal studies were approved by the Institutional Animal Care and Use Committee (IACUC) at the Medical University of South Carolina.

Cigarette Smoke exposure paradigm

Adult mice (n=16) were 6-8 weeks old and aged mice (n=20) were 12 months old when initially exposed to cigarette smoke (CS) for 5 hours/day 5 days/week for 4 months using a Teague TE-10 automated exposure system as described (26, 31). Animals were exposed to 89% sidestream and 11% mainstream CS. To standardize intra-day/batch smoke exposure levels, total suspended particulate matter (TSP) was measured 3 times/day, to maintain a target TSP of 130-150 ppm/m^3 as we have described (21), (31). Exposure at these levels is non-lethal and is associated with carboxyhemoglobin levels between 8% and 12% immediately after exposure, as determined in

venous blood by dual beam spectrophotometry (21). For these experiments, we used 3R4F cigarettes which were purchased from the University of Kentucky, Tobacco Research Institute, and are the reference cigarette of choice in the United States for experiments investigating the effects of Tobacco exposure. The CS exposed mice were subjected to MCAO and treated as described above. Note that in an initial experiment, mice were exposed to 6 months of CS which resulted in 100% mortality by 24 hour after MCAO. Thus the data shown herein was derived from a 4 month CS exposure paradigm.

Neurological Deficit Scoring and Assessment

To assess functional recovery, animals were scored daily by two blinded observers using the neurological deficit score described previously and measured 24 hours after MCAO, just prior to sacrifice (32). Animals received a score of 0 for normal motor function, 1 for torso and contralateral forelimb flexion when lifted by tail, 2 for contralateral circling when held by tail on flat surface, 3 for contralateral leaning when at rest, and 4 for no spontaneous motor activity.

Immunofluorescence Staining and Imaging

Following euthanasia, cardiac perfusion was performed with cooled PBS followed by 4% Paraformaldehyde (PFA) mixed in PBS. Brains were extracted and placed in 4% PFA solution overnight at 4°C. The brains were moved to a new vial with 30% sucrose mixed with 4% PFA and PBS. For tissue cutting, brains were imbedded in optimal cutting temperature (OCT) compound and frozen. At time of cutting, brains were cut in 40 μm coronal sections using a cryostat. The complete brain was collected in 12-well plates and kept in PBS wells until analysis. Brain slices were taken for each brain and were identified by stereometric measurement using a mouse brain atlas and used for immunofluorescent staining as described (8). Slices were washed with PBS and permeabilized using 3% H_2O_2 followed by 0.1% Triton-X in PBS. Slices were blocked in donkey serum (5% in PBS), washed, and incubated with the

primary antibody overnight followed by washing and incubation of the fluorescently tagged secondary antibody. High-resolution imaging was performed using a Zeiss LSM 880 confocal microscope (Zeiss) at 40x zoom with water-media overlay and using the Z-stacking feature of the microscope. Images were deconvoluted using the ZEN 2.5 software (Zeiss) and reconstructed in 3D plane. The primary antibodies used for staining were anti-NeuN (Abcam, Cat. #: ab104225, 1:200) for neurons, anti-Iba1 (Invitrogen, Cat. #: PA5-21274, 1:80) for microglia/macrophages, and anti-MAP2 (Abcam, Cat. #: ab32454, 1:200) for dendrites. Image analysis was performed using both ZEN (Zeiss) and ImageJ (NIH) software. Imaging quantification was performed using unbiased stereology and using full brain slices at similar brain atlas reference coordinates from each group to avoid bias in field selection. Quantification of MAP2 signal was performed by computing the area of MAP2 signal loss in the ipsilateral hemisphere as a percentage of total area of the contralateral hemisphere and reported in percentage. Quantification of Iba1 signal was performed as the percentage of area of increased Iba1 signal density of the area of the contralateral hemisphere and reported in percentage.

Infarct Volume and Survival analysis

Acute infarct volume was estimated using triphenyltetrazolium chloride (TTC) staining of 2 mm thick coronal sections of mice brains as previously described (32). Images were quantified using ImageJ (NIH), and edema-corrected infarct volume was calculated as infarct area x (edema index= area of contralateral hemisphere / area of ipsilateral hemisphere) as described (37). A Kaplan-Meier curve using Mantel-Haenszel log-rank test analysis was performed to compare survival curves.

Statistical Analysis

Statistical analyses were performed using GraphPad Prism 9.0 (GraphPad). Parametric testing was used unless otherwise specified, in the event of Brown-Forsythe test for homogeneity of variance or if normality fails. Histologic analysis for stroke was performed using Chi-squared test. Statistical analyses for infarct and IF analyses were performed using one-way ANOVA test with Bonferroni's correction for multiple comparison. P values below 0.05 were considered significant. Student's t test and Mann Whitney test were used to compare two groups and was used as two-tailed. Pearson correlation coefficients were used to compute correlations.

References:

8. Alawieh A, Adkins DL, Tomlinson S. Acute complement inhibition potentiates neurorehabilitation and enhances tPA-mediated neuroprotection. J Neurosci (2018) 38 (29):6527–45. doi: 10.1186/s12974-015-0464-8

9. Alawieh A, Langley EF, Tomlinson S. Targeted complement inhibition salvages stressed neurons and inhibits neuroinflammation after stroke in mice. Sci Transl Med (2018) 10(441). doi: 10.1038/jcbfm.2010.66

10. Alawieh A A, Zhu H, Yu J, Kindy MS, Atkinson C, Tomlinson S. Modulation of post-stroke degenerative and regenerative processes and subacute protection by sitetargeted inhibition of the alternative pathway of complement. J Neuroinflam. (2015) 12:247. doi: 10.1007/s12975-016-0464-8

21. Woodell A, Coughlin B, Kunchithapautham K, Casey S, Williamson T, Ferrell WD, et al. Alternative complement pathway deficiency ameliorates chronic smoke induced functional and morphological ocular injury. PLoS One (2013) 8(6):e67894. doi:10.1371/journal.pone.0067894

26. Lindbohm JV, Kaprio J, Jousilahti P, Salomaa V, Korja M. Sex, smoking, and risk for subarachnoid hemorrhage. Stroke. (2016) 47(8):1975–81. doi: 10.1186/1471-2202- 15-51

31. Elvington A, Atkinson C, Kulik L, Zhu H, Yu J, Kindy MS, et al. Pathogenic natural antibodies propagate cerebral injury following ischemic stroke in mice. J Immunol (2012) 188(3):1460–8. doi: 10.1159/000353990

32. Jansen O, Macho JM, Killer-Oberpfalzer M, Liebeskind D, Wahlgren N, Group TS. Neurothrombectomy for the treatment of acute ischemic stroke: results from the TREVO study. Cerebrovasc Dis (2013) 36(3):218–25. doi: 10.1161/ STROKEAHA.114.005603

36. Huang Y, Qiao F, Atkinson C, Holers VM, Tomlinson S. A novel targeted inhibitor of the alternative pathway of complement and its therapeutic application in ischemia/reperfusion injury. J Immunol (2008) 181(11):8068–76. doi: 10.4049/ jimmunol.181.11.8068

37. Boyko M, Kuts R, Gruenbaum BF, Tsenter P, Grinshpun J, Frank D, et al. An alternative model of laser-induced stroke in the motor cortex of rats. Biol Proced Online. (2019) 21:9. doi:

10.1186/s12575-019-0097-x
